# 3′mRNA sequencing reveals pro-regenerative properties of *c5ar1* during resolution of murine acetaminophen-induced liver injury

**DOI:** 10.1038/s41536-022-00206-x

**Published:** 2022-01-27

**Authors:** Sina Gonther, Malte Bachmann, Itamar Goren, Arnaud Huard, Andreas Weigert, Jörg Köhl, Heiko Mühl

**Affiliations:** 1 Pharmazentrum frankfurt/ZAFES, General Pharmacology and Toxicology, Faculty of Medicine, Goethe-University Frankfurt am Main, Frankfurt am Main, Germany; 2 Faculty of Medicine, Institute of Biochemistry I, Goethe-University Frankfurt am Main, Frankfurt am Main, Germany; 3 Institute for Systemic Inflammation Research, University of Lübeck, Lübeck, Germany; 4 Division of Immunobiology, Cincinnati Children’s Hospital Medical Center and University of Cincinnati, College of Medicine, Cincinnati, OH USA

**Keywords:** Hepatotoxicity, Cell death and immune response

## Abstract

Murine acetaminophen-induced acute liver injury (ALI) serves as paradigmatic model for drug-induced hepatic injury and regeneration. As major cause of ALI, acetaminophen overdosing is a persistent therapeutic challenge with N-acetylcysteine clinically used to ameliorate parenchymal necrosis. To identify further treatment strategies that serve patients with poor N-acetylcysteine responses, hepatic 3′mRNA sequencing was performed in the initial resolution phase at 24 h/48 h after sublethal overdosing. This approach disclosed 45 genes upregulated (≥5-fold) within this time frame. Focusing on C5aR1, we observed in C5aR1-deficient mice disease aggravation during resolution of intoxication as evidenced by increased liver necrosis and serum alanine aminotransferase. Moreover, decreased hepatocyte compensatory proliferation and increased caspase-3 activation at the surroundings of necrotic cores were detectable in C5aR1-deficient mice. Using a non-hypothesis-driven approach, herein pro-regenerative/-resolving effects of C5aR1 were identified during late acetaminophen-induced ALI. Data concur with protection by the C5a/C5aR1-axis during hepatectomy and emphasize the complex role of inflammation during hepatic regeneration and repair.

## Introduction

Acetaminophen (paracetamol, N-acetyl-p-aminophenol; APAP) is an over-the-counter marketed analgetic broadly used to treat weak-to-moderate pain in clinical practice. Although generally well-tolerated when applied in recommended dosages, APAP is, subsequent to accidental or intentional overdosing, a leading cause of acute liver injury (ALI) world-wide. Of note, delayed presentation/treatment after overdosing is a serious problem that puts patients, including children, at risk for the development of potentially fatal severe ALI^1,2^.

When overdosed, APAP is, to a significant degree, converted to highly reactive N-acetyl-p-benzoquinone imine (NAPQI) by action of hepatocyte cytochrome p450 monooxygenases, foremost CYP2e1 and CYP1a2. Up to a certain level, NAPQI is held in check by glutathione (GSH). Unchecked excessive NAPQI activity, however, initiates a deleterious string of events involving oxidative stress, activation of c-jun N-terminal kinase, mitochondrial dysfunction, and DNA fragmentation—finally culminating in hepatocyte necrosis^3,4^. This initial phase of APAP intoxication can evolve into fatal acute liver failure. Depending on the degree and the individual course of intoxication, the liver may be able to cope with the insult by successfully initiating cellular programs that enable repair and regeneration. Associated compensatory parenchymal hypertrophy is driven by pro-proliferative molecules such as EGF receptor ligands, hepatocyte growth factors, and signal transducer and activator of transcription (STAT)-3-activating cytokines like interleukin (IL)-6^5,6^.

Hepatocyte necrosis in response to APAP is inherently linked to necroinflammation^7^ which is initiated by the release of danger-associated molecular patterns such as high-mobility group box-1 protein (HMGB1)^8^—targeting toll-like receptor (TLR)-4 and receptor of advanced glycation end products, DNA^9^—targeting TLR9, and RNA^10^— targeting TLR3. In terms of function, the role of necroinflammation is perplexing. Whereas inflammatory signaling may possibly contribute to pathogenesis specifically in the early injury phase^11,12^, inflammation is now consistently recognized as firm premise for initiating a successful subsequent resolution/regeneration phase^7^. In that context, upregulation of inflammation-associated protective genes such as IL-10^13^, IL-6^14^, or heme oxygenase-1 (HO-1)^15^ is of particular significance for initiating effective resolution of APAP-induced ALI.

Current pharmacotherapy relies on N-acetylcysteine (NAC) which targets the NAPQI/necrosis-driven injury phase of APAP intoxication. NAC reduces parenchymal necrosis by supporting GSH regeneration and by its capability to directly relief hepatocyte oxidative stress. However, too late onset of NAC therapy (after 24 h) increases the risk to develop a most severe course of intoxication^5^. At that point, liver transplantation is ultimately life-saving for hard-to-treat patients. Presently, there is no therapeutic option available specifically designed to enforce the regeneration phase of intoxication. A notable drug candidate in this context is tissue protective, STAT3-activating IL-22^16,17^. When administered as recombinant protein, this cytokine ameliorates disease in different models of experimental ALI, including APAP-induced ALI^18,19^. Notably, a recent phase 2 clinical trial reports on a beneficial effect of the IL-22-based biopharmaceutical F‐652 in patients with alcoholic hepatitis^20^. Here, we set out to identify hitherto less-investigated pro-resolution/-regeneration pathways that determine the course and outcome of APAP intoxication by using a non-hypothesis-driven 3′mRNA sequencing approach. In these present studies, we identified C5aR1 as an additional parameter that promotes successful resolution of APAP-induced ALI.

## Results

### Gene expression during resolution of APAP-induced ALI as detected by non-hypothesis-driven 3′mRNA sequencing

The course of APAP (300 mg/kg)-induced ALI in C57BL/6J mice shows a brisk necrosis-driven injury phase connecting to the first 24 h of intoxication. At around 24 h after APAP administration, a resolution phase unfolds that aims at restoration of organ function. Starting from the maximum injury at ~24 h, histological liver necrosis has been shown to decline by about 80% during the next 48 h^21^. Based on these observations we set out to identify genes robustly expressed during the initial resolution phase (24–48 h) of APAP-induced ALI in a non-hypothesis-driven 3′mRNA sequencing approach.

**Fig. 1:**
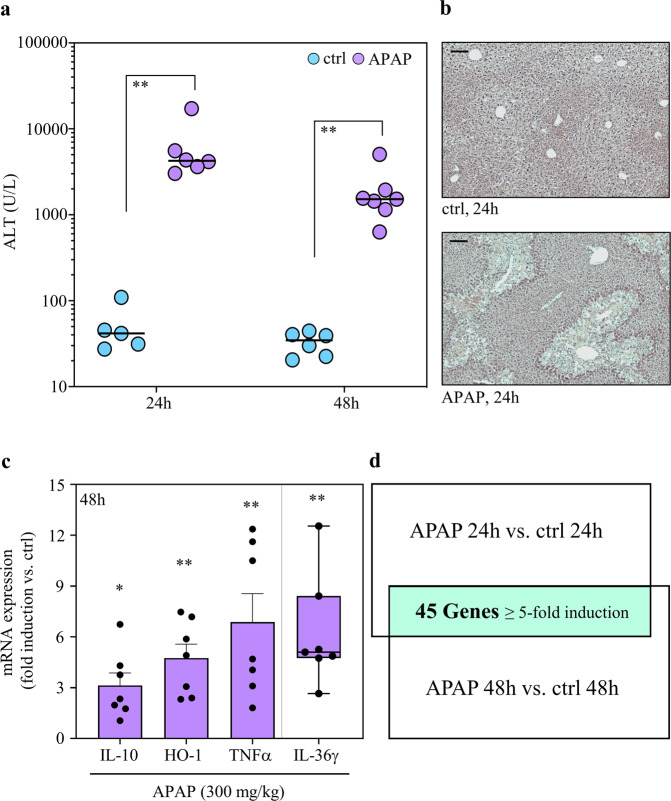
Time-dependent APAP-induced ALI and experimental setup. **a**–**c** C57BL/6J mice received 0.9% NaCl (control) or APAP (300 mg/kg). After the indicated time periods (**a**, 24 h/48 h; **b**, 24 h; **c**, 48 h), liver tissue and sera were analyzed. **a** Liver damage by serum ALT (*n* = 5 and *n* = 6 for 24 h and 48 h control groups, *n* = 6 and *n* = 7 for 24 h and 48 h APAP groups; **p < 0.01). **b** Liver sections (H&E) at 24 h. One representative of control (*n* = 6) and APAP (*n* = 7) groups; **c** Hepatic mRNA (derived from mice of (**a**) at 48 h) for IL-10, HO-1, TNFα, and IL-36γ (real-time PCR). Target mRNA normalized to GAPDH is shown as fold-induction compared to control (*n* = 6 for control, *n* = 7 for APAP; *p < 0.05, **p < 0.01 vs. control). **d** Hepatic RNA derived from mice shown in (**a**) (plus 1x control/1x APAP at 24 h—no ALT data available for these two mice) were pooled (per experimental condition) in equal shares and evaluated for gene expression by MACE. After applying a threshold of ‘5-fold gene induction’ (APAP vs. control at the same time point), 45 genes were identified to be consistently upregulated at 24 h/48 h (within the resolution phase of APAP intoxication). A schematic of the experimental setup and the results is given. Statistical analysis on raw data: **a** Mann–Whitney-U-test, raw data are shown; **c** Student’s *t*-test (IL-10, HO-1, TNFα), data are shown as means ± s.e.m.; Mann–Whitney-U-test (IL-36γ), data are shown as box-plots. Scale bars: 50 μm.

For that purpose, C57BL/6J mice were either kept as vehicle-treated control or treated with APAP at the non-lethal dosage of 300 mg/kg. High serum alanine aminotransferase (ALT) activity, a surrogate marker of liver necrosis, confirmed efficient induction of ALI at 24 h after APAP administration. During the following 24 h, ALT levels decreased by 64.3%. This decline is consistent with previous data^21^ but did not reach statistical significance in the set of experiments performed (Fig. 1a). Figure 1b displays representative histological liver damage at 24 h after APAP administration along with respective vehicle-treated controls. Next, expression of selected genes previously connected to protection/resolution from APAP-induced ALI was investigated, namely that of IL-10^6,7^, HO-1^22^, TNFα^6,7,23^, and IL-36γ^24^. For that analysis, hepatic RNA derived from mice shown in Fig. 1a (at 48 h) was investigated. As expected, increased expression of these parameters was detectable in the resolution phase of APAP-induced ALI as compared to the vehicle-treated control group (Fig. 1c).

**Fig. 2:**
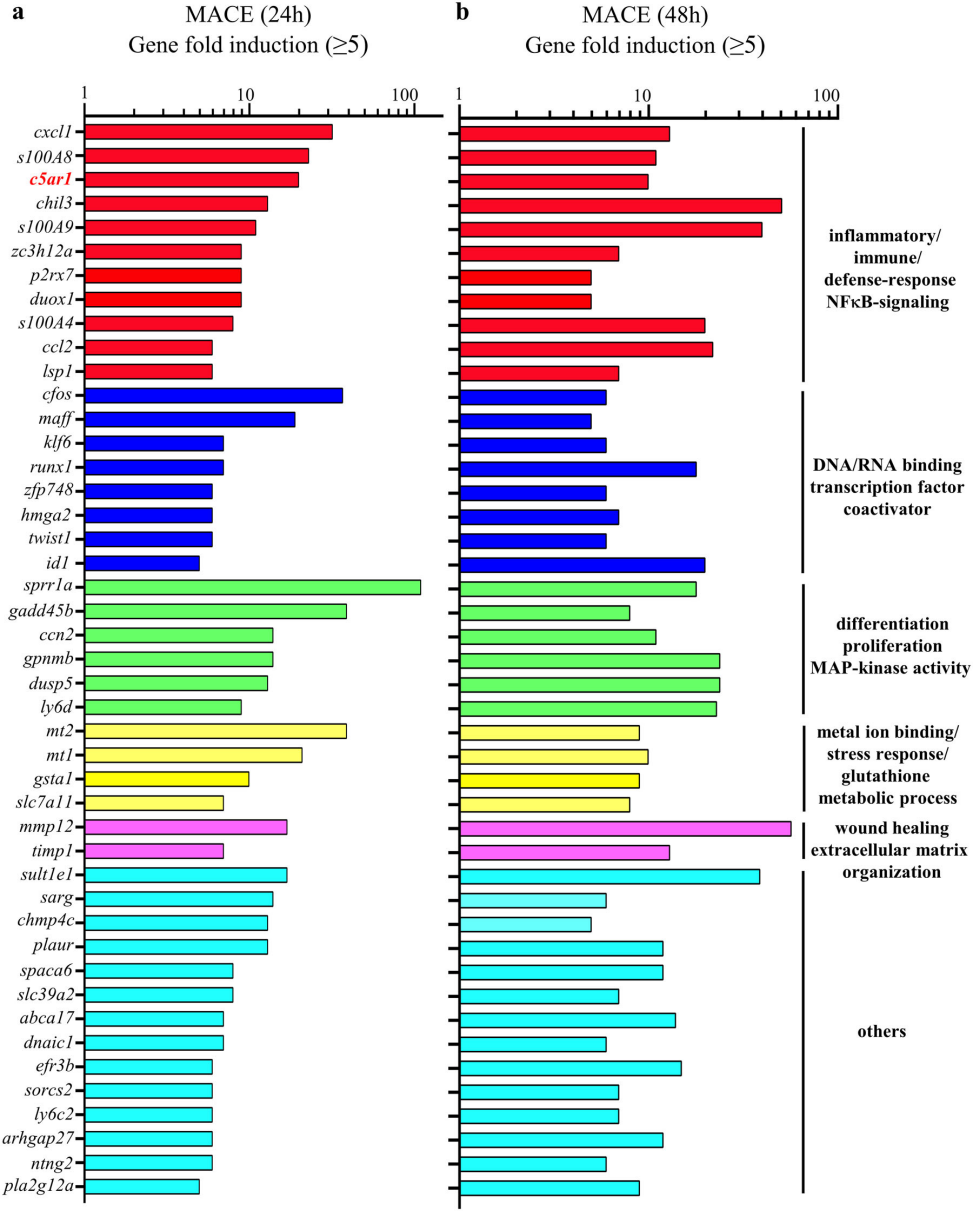
3′mRNA sequencing by MACE at time points 24 h/48 h after APAP administration. Analysis disclosed a 24 h/48 h intersection with shared robust induction of 45 genes (threshold: ≥5-fold gene induction by APAP treatment (300 mg/kg) vs. control) in the resolution phase of intoxication. Induction of these 45 genes is depicted at the 24 h (**a**) and the 48 h (**b**) time points. Genes were grouped into selected GO molecular functions/biological processes based on the UniProt database at https://www.uniprot.org.

After having characterized the current experimental setup, hepatic RNA populations derived from mice shown in Fig. 1a (plus 1x control/1x APAP at 24 h—no ALT data available for these two mice) were used for 3′mRNA sequencing. For that purpose, hepatic RNA specimens from all mice of the 4 groups (vehicle-treated at 24/48 h, *n* = 6; APAP treated at 24/48 h, *n* = 7) were pooled (per group) at equal shares. Thereafter, pooled RNA underwent analysis by 3′mRNA sequencing (massive analysis of cDNA ends, MACE). To identify those mRNAs that were most consistently expressed during the initial resolution phase, we focused on gene expression at the intersection between 24 and 48 h and applied a robust threshold of ‘5-fold gene induction’ compared to vehicle-treatment. As depicted in Fig. 1d, 45 differentially expressed genes were identified by this experimental approach. Genes categorized in ‘GO molecular functions/biological processes terms’ (https://www.uniprot.org)^25^ are listed based on their ‘MACE gene fold-induction’ values at time points 24 h/48 h. Two prominent larger GO categories covered by gene expression during this initial resolution phase of APAP-induced ALI were ‘inflammatory/immune/defense responses and NF-κB signaling’ as well as ‘differentiation/proliferation/MAPK activity’ (Fig. 2).

Proceeding from these data, expression of selected genes identified by MACE was verified by real-time PCR of individual RNA samples used for pooling. Supplementary Figure 1 shows mRNA data for *s100a8* (a), *pla2g12a* (b), and *dusp5* (c) at 24 h (left panels) and 48 h (right panels). Upregulation of mRNA expression for *ccn2*, *gadd45b*, *ccl2*, *mt1*, *mt2*, and *mmp12* was confirmed for the time point 30 h after administration of APAP (Supplementary Fig. 1d). Altogether, gene induction as detected by MACE was confirmed by real-time PCR for every gene verified.

### *c5ar1* gene expression is upregulated during APAP-induced ALI

**Fig. 3:**
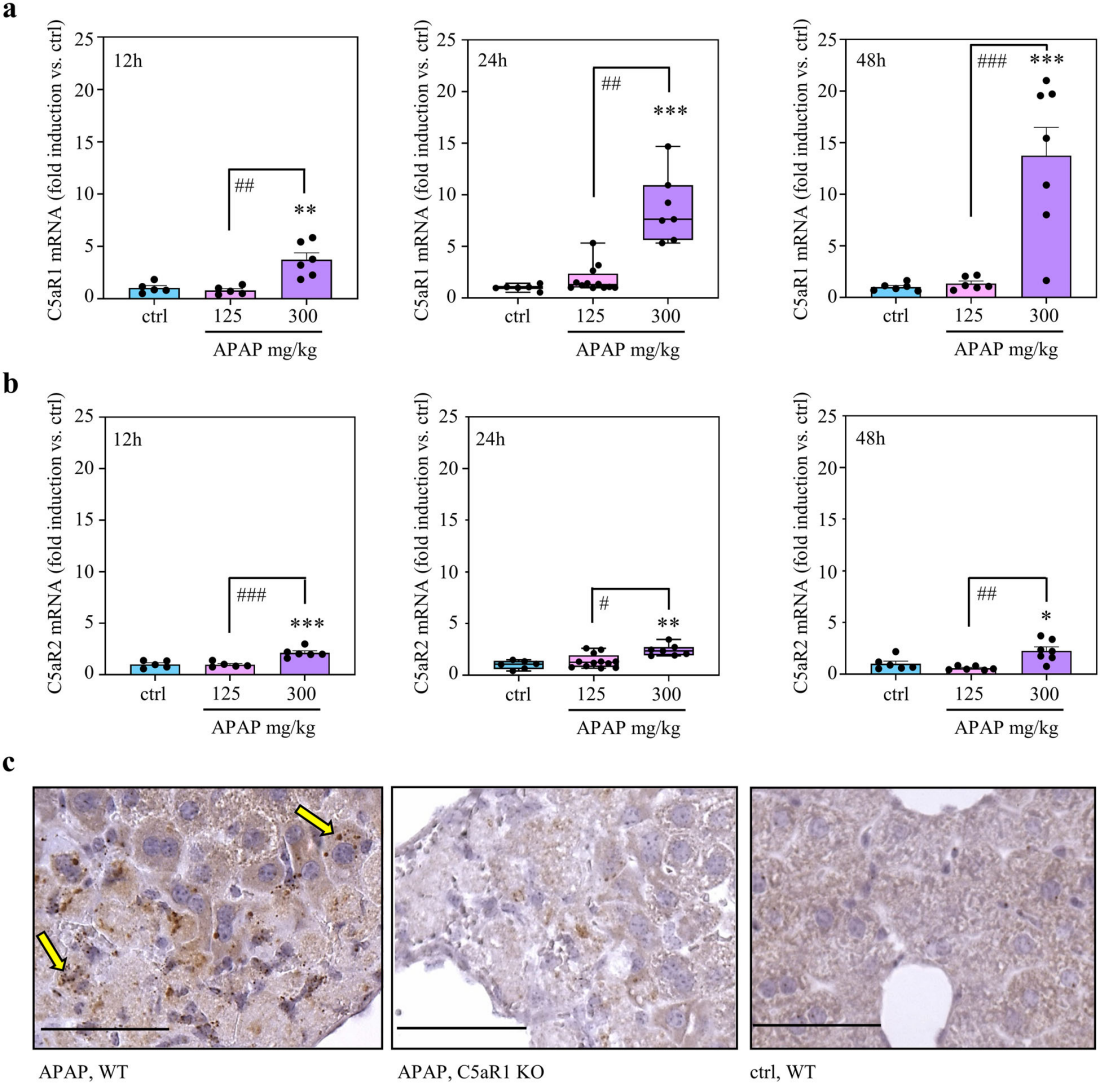
Gene induction of *c5ar1* during APAP-induced ALI. **a**, **b** C57BL/6J mice received 0.9% NaCl (12 h, *n* = 5; 24 h/48 h, *n* = 6), APAP at 125 mg/kg (12 h, *n* = 5; 24 h, *n* = 12; 48 h, *n* = 6), or APAP at 300 mg/kg (12 h, *n* = 6; 24 h/48 h, *n* = 7). After the indicated time points, hepatic RNA was isolated. For controls and APAP at 300 mg/kg (24 h/48 h), individually analyzed RNA populations were the same that were used for pooling in MACE. Hepatic C5aR1 (**a**) and C5aR2 (**b**) mRNA was determined by real-time PCR at 12 h (left panel), 24 h (middle panel), or 48 h (right panel) after APAP administration. C5aR1 and C5aR2 mRNA normalized to GAPDH is shown as fold-induction compared to control of the same time point (*p < 0.05, **p < 0.01, ***p < 0.001 vs. control at the same time point; ^#^*p* < 0.05, ^##^*p* < 0.01, ^###^*p* < 0.001). Statistical analysis on raw data: (**a**, **b** middle panel) Kruskal–Wallis test with Dunn’s post hoc test, data are shown as box-plots; (**a**, **b** left/right panel) ANOVA with Bonferroni post hoc test, data are shown as means ± s.e.m. **c** C57BL/6J C5aR1-deficient mice and their wild-type counterparts (*n* = 4 per group) received 0.9% NaCl (control) or APAP (300 mg/kg). After 30 h, hepatic C5aR1 mRNA was analyzed by in situ hybridization (RNAscope). Representative specimens are shown. Specimens underwent processing in parallel. C5aR1 positivity shows as fine-grained dark-brownish dots. C5aR1 mRNA expressing hepatocytes are exemplarily indicated as yellow arrows. Wild-type mice, WT; knockout mice, KO. Scale bars: 50 μm.

MACE analysis identified *c5ar1*^26^ as robustly expressed component of the inflammatory gene cluster upregulated during resolution of APAP-induced ALI (Fig. 2). Because this receptor for the C5a anaphylatoxin^27–29^ is a key parameter in diverse liver diseases^30^ and has been shown to support recovery from murine hepatectomy^31,32^ and CCl_4_-induced liver injury^33^, we focused on the role of C5aR1 during the resolution phase of APAP-induced ALI. For that purpose, RNA populations used in MACE analysis were individually evaluated for *c5ar1* gene expression. Interestingly, *c5ar1* gene induction steadily increased during the course of APAP-induced ALI (Fig. 3a). In contrast, mRNA coding for the second C5aR, namely C5aR2^34^, was well detectable but increased only marginally without apparent time dependence (Fig. 3b). Based on raw data (24 h, target gene expression/glyceraldehyde-3-phosphate dehydrogenase (GAPDH) given as median), hepatic C5aR2 mRNA was low as compared to C5aR1—under control conditions and upon APAP (control (*n* = 6): C5aR1 1.0 × 10^−3^, C5aR2 0.6 × 10^−3^; APAP (*n* = 7): C5aR1 7.9 × 10^−3^, C5aR2 1.3 × 10^−3^).

Of note, analysis of mRNA expression shown in Fig. 3 and Supplementary Fig. 1a–c suggested that gene induction under the influence of APAP (Fig. 2), correlates with significant liver necrosis. Specifically, administration of grossly subtoxic 125 mg/kg APAP (median serum ALT at 24 h (*n* = 12) and 48 h (*n* = 6), 72.0 U/L and 44.8 U/L, respectively) failed to mediate induction of genes such as *c5ar1*, *s100a8*, *dusp5*, and *pla2g12a*.

Previous reports demonstrate scattered ‘physiological’ expression of C5aR1 in murine Kupffer cells^35,36^. Interestingly, healthy murine^35,37^, rat^38,39^, and human^40^ hepatocytes lack detectable C5aR1 protein expression. In situ hybridization by RNAscope analysis revealed *c5ar1* gene induction in hepatocytes under the influence of APAP-induced necroinflammation (Fig. 3c). Specifically, we observed upregulation of hepatocyte C5aR1 mRNA visible as fine-grained dark-brownish dots in livers of APAP (300 mg/kg)-treated wild-type mice (Fig. 3c, left panel—exemplarily indicated by yellow arrows). Besides background staining, we did not observe those specific signals in specimens derived from corresponding APAP-treated C5aR1-deficient mice (Fig. 3c, middle panel) or from those derived from corresponding 0.9% NaCl-treated wild-type mice (Fig. 3c, right panel).

### C5aR1-deficient mice display aggravation of disease in late APAP-induced ALI

**Fig. 4:**
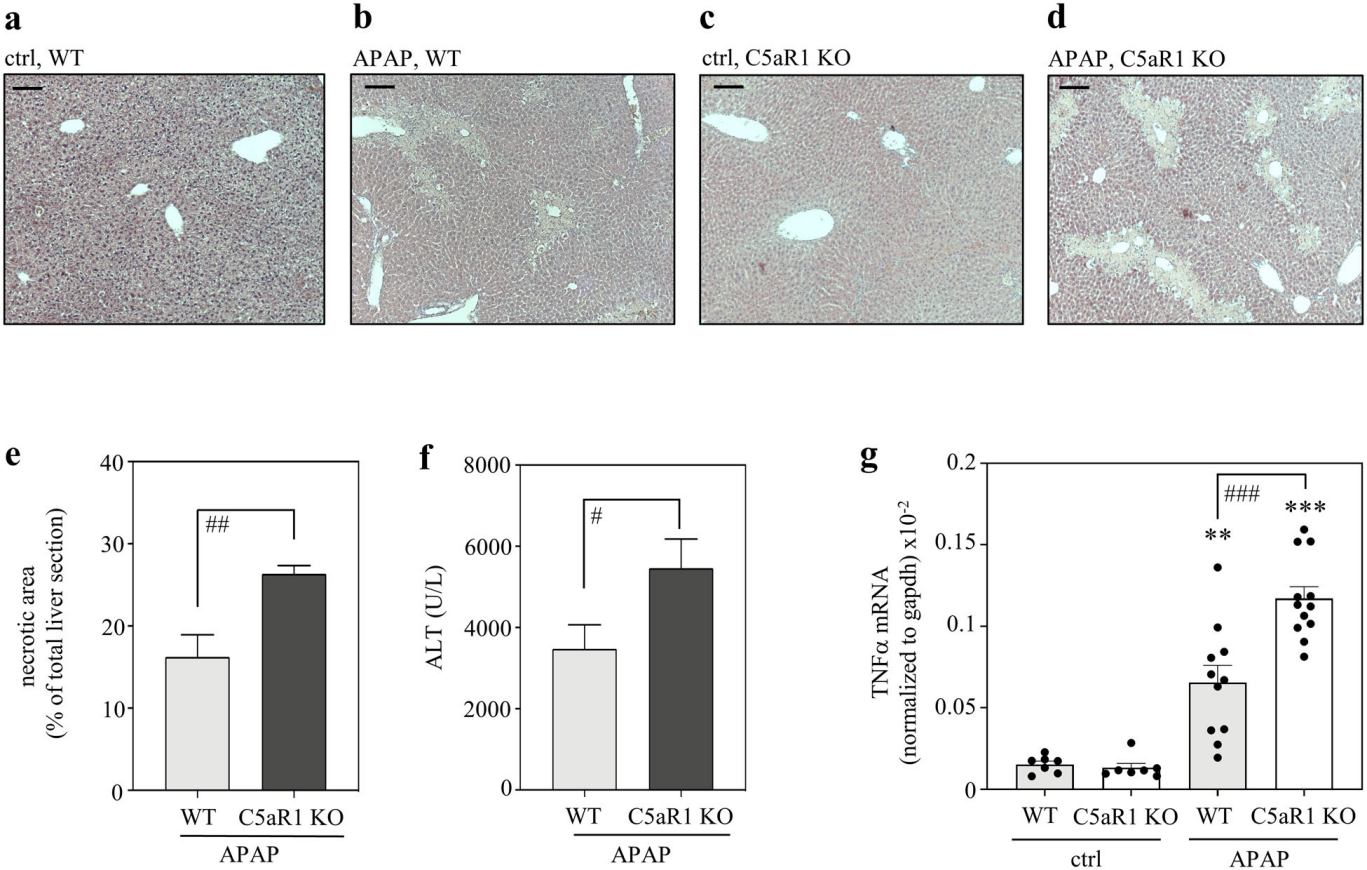
C5aR1-deficient mice display aggravated APAP-induced ALI. C57BL/6J C5aR1-deficient mice and their wild-type counterparts received 0.9% NaCl (control groups) or APAP (300 mg/kg). After 30 h, mice were sacrificed and liver tissue and sera were analyzed. **a**–**d** Representative liver sections (H&E staining) of vehicle-treated control (wild-type (*n* = 5), C5aR1-decicient (*n* = 5)) or APAP-treated (wild-type (*n* = 11), C5aR1-deficient (*n* = 12)) mice. **e** Analysis by BZ-II analyzer software of necrotic areas in H&E-stained liver sections from APAP-treated mice (wild-type, *n* = 11; *c5ar1*^−/−^, *n* = 12; ^##^*p* < 0.01). **f** Liver damage as detected by serum ALT (wild-type, *n* = 11; *c5ar1*^−/−^, *n* = 12; ^#^*p* < 0.05). **g** Hepatic mRNA was isolated from 0.9% NaCl-treated control groups (each *n* = 7), from APAP-treated C5aR1-deficient mice (*n* = 12), and from their APAP-treated wild-type counterparts (*n* = 11). TNFα mRNA, determined by real-time PCR, was normalized to GAPDH and is shown as absolute values (**p < 0.01, ***p < 0.001 vs. control of the respective genotype; ^###^*p* < 0.001). Statistical analysis on raw data: **e**, **f** Student’s *t*-test, data are shown as means ± s.e.m. **g** ANOVA with Bonferroni post hoc test, data are shown as means ± s.e.m. Wild-type mice, WT; knockout mice, KO. Scale bars: 50 μm.

In order to address the role of C5aR1 during resolving APAP-induced ALI, C57BL/6J mice with a general C5aR1 knockout^41^ and their respective wild-type counterparts were exposed to the non-lethal APAP overdosage of 300 mg/kg. Thereto, a 30 h observation period was selected which covers the initial resolution phase after intoxication. Figure 4a–d shows representative histological sections from vehicle- and APAP-treated mice of both genotypes. Quantification of hepatic necrotic areas (e) and serum ALT (f) revealed aggravated APAP intoxication in C5aR1-deficient mice (mean serum ALT in vehicle-treated control wild-type and knockout mice, 54.9 and 63.1 U/L (*n* = 7 per group); necrotic area in vehicle-treated animals of both genotypes, not detectable). Hepatic TNFα expression is upregulated during APAP-induced ALI^19^ and can be regarded as an additional surrogate of necroinflammation. In accord with pronounced necrosis seen in C5aR1-deficient mice, we likewise observed enhanced hepatic TNFα expression in these same animals (Fig. 4g). These findings suggested that activation of C5aR1 supports successful resolution of APAP-induced ALI.

In addition, effects of C5aR1 deficiency were evaluated at the earlier 6 h (Supplementary Fig. 2a–c) and 24 h (Supplementary Fig. 2d–f) time points after APAP administration. In contrast to the 30 h time point, those earlier time points did not display enhanced hepatic necrosis in C5aR1-deficient mice. Of note, the 24 h time point marks the peak of hepatic injury and the onset of tissue recovery in this model^21^. The time course of hepatic damage (Supplementary Fig. 2g) in fact revealed that disease resolution is active between 24 h and 30 h after APAP administration. Specifically, analysis of this 6 h time frame in wild-type mice revealed a significant drop in histological necrosis from 27.7% (at 24 h) to 16.2% (at 30 h). Altogether, these data indicate that protective properties of C5aR1 during APAP-induced ALI specifically support the ongoing resolution in the later phase of intoxication.

**Fig. 5:**
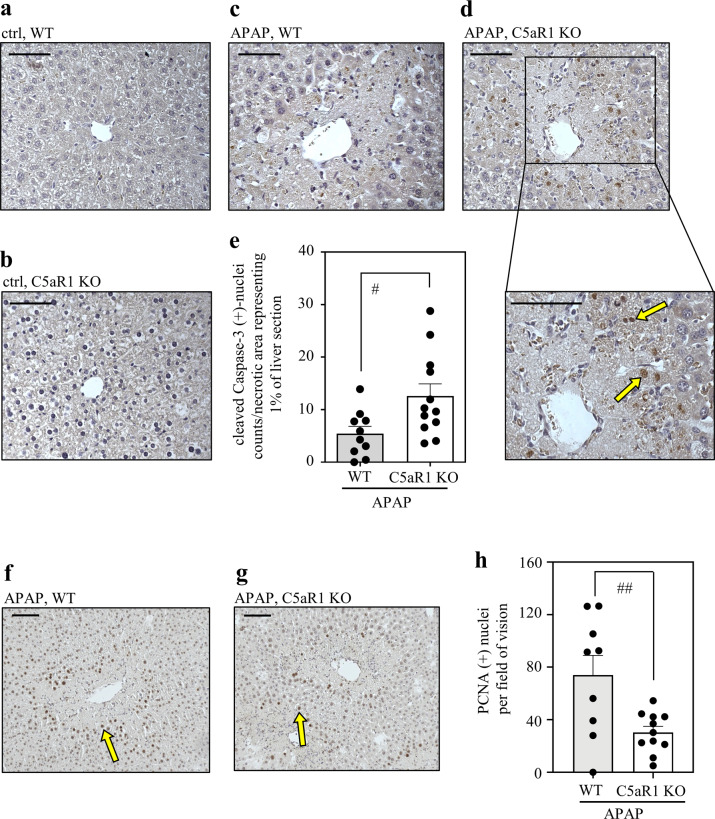
C5aR1-deficient mice display dysregulated hepatocyte caspase-3 activation and impaired compensatory proliferation in the regeneration phase of APAP-induced ALI. **a**–**d** C57BL/6J C5aR1-deficient mice and their wild-type counterparts received 0.9% NaCl (control groups) or APAP (300 mg/kg). After 30 h, liver tissue was evaluated for the presence of active cleaved caspase-3 by immunohistochemistry. Representative results are shown for vehicle-treated wild-type (**a**, *n* = 7), vehicle-treated C5aR1-deficient (**b**, *n* = 7), APAP-treated wild-type (**c**, *n* = 10), and APAP-treated C5aR1-deficient mice (**d**, *n* = 12). **e** Quantification of cleaved caspase-3-positive nuclei detectable in APAP-treated wild-type (*n* = 10) and C5aR1-deficient mice (*n* = 12). Positive nuclei (almost exclusively found within/adjacent to necrotic regions; exemplarily indicated by yellow arrows) were counted and related to the percentage of necrotic area in the respective liver segment (^#^*p* < 0.05). Statistical analysis on raw data: **e** Student’s *t*-test, data are shown as means ± s.e.m. **f**–**h** C5aR1-deficient mice and their wild-type counterparts received APAP at 300 mg/kg. After 30 h, mice were sacrificed and liver tissue was analyzed. **f**, **g** Nuclear PCNA protein was determined by immunohistochemistry. Representative results are shown for APAP-treated wild-type mice (**f**, *n* = 9) and C5aR1-deficient mice (**g**, *n* = 11). **h** PCNA-positive nuclei (exemplarily indicated by yellow arrows) were counted per field of vision and are shown as means ± s.e.m. (^##^*p* < 0.01). Statistical analysis on raw data: Student’s *t*-test. Wild-type mice, WT; knockout mice, KO. Scale bars: 50 μm.

Since C5aR1 has been connected to anti-apoptotic signaling in proliferating hepatocytes during murine hepatectomy^31^, activation of caspase-3 was evaluated under aforementioned experimental conditions. For that purpose, immunohistochemistry specifically addressing active caspase-3 was performed in order to determine caspase-3-positive nuclei in hepatic tissues exposed to APAP. Notably, nuclear translocation of active caspase-3 is regarded as a hallmark of apoptosis^42^. Whereas vehicle-treated wild-type and C5aR1-deficient control mice did not show any hepatic caspase-3 activation (Fig. 5a, b), modest nuclear staining was detectable in hepatocytes at necrotic areas from wild-type mice undergoing APAP intoxication (Fig. 5c). Hepatocyte nuclear caspase-3-staining of APAP-treated C5aR1-deficient mice (Fig. 5d) was significantly higher in numbers (Fig. 5e) and evidently stronger. Of note, intense nuclear staining of caspase-3 was located foremost to the outer areas/surroundings of necrotic regions (Fig. 5d) which must be regarded as heavily exposed to inflammatory signals. These observations suggest that lack of C5aR1 may compromise resolution from APAP intoxication by enhancing apoptosis of those hepatocytes in the surroundings of necrotic cores that survived the initial exposure to pro-necrotic APAP/NAPQI.

Finally, hepatocyte proliferation was determined by immunohistological evaluation of proliferating cell nuclear antigen (PCNA)-positive hepatocyte nuclei^14^. As shown in Fig. 5f–h, C5aR1-deficient mice displayed significantly diminished compensatory hepatocyte proliferation which is in agreement with previous observations of a pro-proliferative role for C5aR1 in murine hepatectomy^31,32^ and CCl_4_-induced liver damage^33^.

## Discussion

In the current study, non-hypothesis-driven 3′mRNA sequencing by MACE disclosed a group of 45 genes that are consistently expressed at time points 24 h/48 h after administration of APAP and thus can be considered as gene candidates potentially affecting resolution from intoxication. Some of these genes have already been connected to protection in the context of APAP intoxication, namely *ccl2*^43^, *gadd45b*^44^, *mt1/2*^45^, and *mmp12*^46^. Real-time PCR performed herein indeed confirmed increased mRNA expression of these 5 genes during the initial resolution phase of APAP-induced ALI.

In subsequent work, we focused on C5aR1 which is generally regarded as key receptor for the anaphylatoxin C5a^27,28^. In fact, complement activation detected by C3 cleavage (known to result in C5a generation)^47^, can be observed during murine^47^ and human^48^ APAP-induced ALI. However, besides regular C3-dependent activation, strong necroinflammation as seen in APAP intoxication^7^ is in addition capable of directly generating C5a by non-canonical (C3-independent) pathways that depend on neutrophil and/or macrophage activation and associated protease release^28,49^. Herein, we identified by MACE robust upregulation of hepatic *c5ar1* gene expression during the regeneration phase of APAP-induced ALI. Subsequent in situ hybridization by RNAscope verified hepatocyte C5aR1 expression in response to APAP intoxication.

In order to study the function of C5aR1 within the resolution phase of APAP-induced ALI, C5aR1 knockout mice were investigated 30 h after APAP application. C5aR1-deficient mice showed significantly aggravated intoxication which was evident at the level of histological damage and augmented serum ALT. In contrast, exacerbation of liver damage was not detectable in C5aR1-deficient mice at the earlier 6 h and 24 h time points of intoxication. This observation proved that aggravation of liver injury by C5aR1 deficiency connects to the later regeneration/resolution phase of APAP intoxication.

Protective properties of C5aR1 in late APAP-induced ALI concur with its pro-regenerative/proliferative characteristics that were previously observed in murine CCl_4_-induced liver injury^33^ and rodent hepatectomy^31,32,39^. In fact, we also observed significantly reduced compensatory hepatocyte proliferation in C5aR1-deficient mice undergoing APAP intoxication. Moreover, during murine hepatectomy, C5aR1 curbs caspase-3-dependent apoptosis of regenerating hepatocytes^31^. Non-myeloid (likely hepatocyte) C5aR1 expression likewise limits hepatocyte caspase-3 activation and apoptosis after chronic ethanol feeding^50^. Whereas the decisive mode of cellular demise during the injury phase of APAP-induced ALI is (programmed) necrosis^4^, we herein confirm the aforementioned connection between C5aR1 and the modulation of hepatocyte apoptosis with respect to the later stage of APAP intoxication. Specifically, we demonstrate that lack of C5a/C5aR1 signaling increased hepatocyte caspase-3 activation and thus apoptosis during the resolution phase of intoxication. Those apoptotic hepatocytes were located mostly in the surroundings of necrotic cores which are considered highly inflammatory tissue regions. Of note, C5aR1 is supposed to augment nuclear factor-κB activation in the regenerating liver^32^ and in hepatocytes^51^ and, by supporting cell viability, this transcription factor plays a crucial role for the hepatocytes’ decision between proliferation and apoptosis^52^. C5aR1 in addition belongs to the group of G protein-coupled receptors and, among others, initiates signaling via Ca^2+^, the phosphatidyl 3-kinase/Akt-, and the mitogen-activated protein kinase/extracellular signal-regulated kinases (ERK)-1/2-pathways^28^. In fact, all these three signaling pathways are supposed to promote liver regeneration^53,54^. The present data thus indicate dysfunctional resolution of APAP-induced ALI as a consequence of insufficient C5aR1 signaling which diminishes regenerative hepatocyte proliferation and promotes apoptosis of distinct hepatocytes in the surroundings of necrotic cores that survived the initial necrosis-activating exposure to APAP/NAPQI.

Pro-regenerative properties of C5a in APAP-induced ALI may result from direct activation of C5aR1 on hepatocytes. Herein we demonstrate that hepatocyte C5aR1 expression is in fact induced during APAP intoxication. This path has been previously proposed in the context of murine hepatectomy^32^. Whereas C5aR1 protein is barely detectable in hepatocytes of healthy mice^35,37^ and rats^38,39^, its expression is likewise upregulated during rat hepatectomy^39^, murine LPS/D-galactosamine-induced fulminant liver injury^35^, and murine sepsis^37^. Expression of C5aR1 by hepatocytes during these inflammatory conditions is supposed to be mediated by Kupffer cell/macrophage-derived IL-6^55^, a cytokine that is evidently upregulated in response to APAP intoxication^14^. Interestingly, induction of hepatocyte C5aR1 in rat hepatectomy is specifically assigned to regenerating hepatocytes^39^. Besides that, indirect immunoregulatory/-modulatory properties of the C5a/C5aR1-axis may likewise promote resolution from APAP toxicity: (a) C5aR1 activation enhances production of pro-regenerative/anti-apoptotic IL-6^6,17^ by Kupffer cells^56^; (b) pro-migratory properties of the C5a/C5aR1-axis should support hepatic infiltration by neutrophils which, through removing cell debris, is considered a predominantly protective cell type in this context^7^; (c) C5aR1 activation impairs TLR4-dependent production of IL-12 and IL-23^57^. This latter point is particularly interesting because IL-23^8^ and IL-12-downstream IFNγ^58^ are regarded pathogenic in APAP intoxication and several studies report on amelioration of intoxication in TLR4-deficient mice^59–61^. (d) Finally, the C5a/C5aR1-axis promotes liver fibrosis^62^. Whereas uncontrolled fibrosis is certainly pathogenic in chronic disease, a fine-tuned/regulated fibrotic response is supposed to contribute to repair in acute liver injury.

It must, however, be noted that the role of C5a in APAP intoxication is likely multifaceted. Particularly under conditions of insufficient C5aR1, C5a should effectively activate its alternative receptor C5aR2. Notably, C5aR2 activation has been shown to promote release of HMGB1^63^ which is evidently pathogenic in APAP-induced ALI^8^. It is furthermore noteworthy that lack of C3 biological function was associated with amelioration of APAP-induced ALI^47,64^. Yet, this observation is likely unrelated to C5a receptor functions. In fact, during chronic and acute hepatitis, including APAP-induced ALI, activation of the cytolytic membrane attack complex (C5b-9) is detectable on hepatocytes at areas of necrosis^48,65,66^. This C5aR1/C5aR2-independent process should contribute to hepatocyte cell death and thus pathogenesis of necroinflammation.

Taken together, non-hypothesis-driven 3′mRNA sequencing identified a beneficial role for C5aR1 in the resolution phase of APAP-induced ALI. Our data reinforce the concept that inflammation and repair are intertwined parameters of pathophysiology. To translate this knowledge into therapeutic strategies that specifically address repair and regeneration is a key task of current research.

## Methods

### Animal studies

All animal experiments (male C57BL/6J mice, 9–12 weeks old, maintained/breed at MfD Diagnostics, Wendelheim, Germany) were carried out in accordance with the recommendations of the Animal Protection Agency of the Federal State of Hessen (Regierungspräsidium Darmstadt, Germany) and were approved by the Regierungspräsidium Darmstadt (references V54-19c20/15-FU1190 and -FU1230). All mice were maintained in type II-long-IVC under a 12 h light-dark cycle with access to food and water *ad libitum* (with the exception of a 10 h overnight fasting period (with free access to water) before APAP (or vehicle) administration, for details see below). C57BL/6J Mice displaying a general *c5ar1* knockout (C5ar1^−/−^)^41^ and their specific wild-type controls (used in Figs. 3c, 4 and 5, Supplementary Fig. 2 (wild-type and knockout mice), and in Supplementary Fig. 1d (wild-type mice)) were originally obtained from the Institute for Systemic Inflammation Research (University of Lübeck, Lübeck, Germany). Both strains entered the Lübeck animal facility by embryo transfer and were since then maintained under equal housing conditions. Accordingly, microbiome differences between these two strains due to housing conditions are unlikely. Genetic identity *c5ar1* knockout and their specific wild-type controls were verified by PCR for every mouse included in the study. After completion of the experimental protocol, mice underwent short isoflurane (Abbott, Wiesbaden, Germany) anesthesia and were sacrificed by cervical dislocation. Blood was taken from the retroorbital venous plexus with serum stored at −80 °C. For tissue processing, livers were perfused with PBS. Specimens were, thereafter, incubated in 4.5% buffered formalin overnight for histological analysis on paraffin-embedded sections. For analysis of mRNA expression, specimens were snap-frozen and stored at −80 °C.

### APAP-induced ALI

Unfasted mice show augmented variability and female mice attenuated toxicity in models of APAP-induced ALI^67,68^. Thus, male mice fasted overnight (10 h) were used herein—which is in accord with the consensus in the field. Murine APAP-induced liver injury was performed as previously described^19^. Briefly, fasted male mice obtained i.p. injections of warm (body tempered) 0.9% NaCl solution (B. Braun, Melsungen, Germany) or of either 125 mg/kg or 300 mg/kg APAP (Sigma-Aldrich (Taufkirchen, Germany) that was dissolved in warm 0.9% NaCl (Sigma-Aldrich). Mice that obtained NaCl solution are referred to as control mice. Thereafter, mice had free access to food and water. After 6, 12, 24, 30, or 48 h, experiments were terminated.

### Evaluation of liver injury

Serum ALT activity was determined according to the manufacturer’s instructions (Reflotron; Roche Diagnostics GmbH, Mannheim, Germany). Paraffin-embedded liver sections (4 μm) were stained with H&E. Histopathological liver injury/necrosis was quantified by Keyence BZ-II Analyzer software (Neu-Isenburg, Germany). Necrotic area is expressed as (% of total liver section).

### Evaluation of gene expression by real-time PCR

Total RNA, isolated by Tri-Reagent (Sigma-Aldrich) was transcribed using random hexameric primers (Qiagen, Hilden, Germany) and Moloney virus reverse transcriptase (Thermo Fisher Scientific). RNA isolates were generally digested with RNase-free DNase I (Roche Diagnostics GmbH, Mannheim) before reverse transcription. During real-time PCR, changes in fluorescence were caused by the Taq polymerase degrading the probe that contains a fluorescent dye (GAPDH): VIC, all other probes: FAM; Thermo Fisher Scientific). Pre-developed reagents: *gapdh* (4352339E), *c5ar1* (Mm00500292_s1), *c5ar2* (Mm01267981_s1), *hmox1* (Mm00516005_m1), *s100a8* (Mm00496696_g1), *dusp5* (Mm01266106_m1), *pla2g12a* (Mm01316982_m1), *tnf* (Mm00443258_m1), *gadd45b* (Mm00435123_m1), *ccn2* (Mm01192933_g1), *ccl2* (Mm00441242_m1), *mt1* (Mm00496660_g1), *mt2* (Mm00809556_s1), *mmp12* (Mm00500554_m1), *il10* (Mm01288386_m1), and *il36g* (Mm00463327_m1). Assay-mix was from Nippon Genetics (Düren, Germany). Real-time PCR was performed on 7500 Fast Real-Time PCR System and QuantStudio 3 Sequence Detector (Thermo Fisher Scientific): One initial step at 95 °C (2 min) was followed by 40 cycles at 95 °C (5 s) and 62 °C (30 s). Detection of the dequenched probe, calculation of threshold cycles (Ct values), and data analysis were performed by the Sequence Detector software. Relative changes in mRNA expression compared to unstimulated control and normalized to GAPDH were quantified by the 2^−ddCt^ method or were shown as 2^−dCT^ (expression relative to GAPDH, absolute values).

### Immunohistochemistry

For detection of hepatic cleaved (active) caspase-3 and nuclear PCNA paraffin-embedded liver sections (4 μm) were used. Sections were deparaffinized and unmasked by heat treatment (Dako Target Retrieval Solution, Dako Glostrup, Denmark). Thereafter, sections were incubated overnight at 4 °C with a rabbit polyclonal anti-cleaved-caspase-3 antibody (DCS Innovative Diagnostik-Systeme (CI752C002), Hamburg, Germany) or a rabbit monoclonal anti-PCNA antibody (Cell Signaling, (13110), Frankfurt, Germany). Antibodies were diluted in an antibody dilution reagent obtained from DCS Innovative Diagnostik-Systeme. Histofine Simple Stain Max Po Anti-Rabbit (Nichirei Biosciences Inc., Tokyo, Japan) and DAB Substrate Kit for Peroxidase (Vector Laboratories, Burlingame, CA, USA) were used for detection of cleaved (active) caspase-3 and PCNA. Cleaved caspase-3 sections were counterstained using hematoxylin (AppliChem GmbH, Darmstadt, Germany). Caspase-3-positive nuclei were counted in blinded manner and related to the total percentage of necrotic area found in the same liver segment. One section was evaluated per mouse (wild-type, *n* = 10; knockout, *n* = 12). Data are shown as (counts/necrotic area representing 1% of the given liver section). PCNA-positive nuclei were counted in blinded manner. Depicted values are derived from four randomly chosen fields of vision (objective size 20×) taken from each (same) liver segment (wild-type, *n* = 9; C5aR1-deficient mice, *n* = 11). Data are shown as PCNA-positive nuclei per field of vision.

### 3′mRNA sequencing

RNA specimens were digested by DNase I recombinant RNase-free Kit (Roche Diagnostics) according to manufacturer’s instructions. After quality check by electrophoresis, samples of pooled RNA were generated as outlined in the “Results” section. Two micrograms of RNA from each pool was used for analysis by 3′mRNA sequencing. Massive Analysis of cDNA ends (MACE) stands for a 3′mRNA sequencing approach using Illumina reads of fragment that are derived from 3′mRNA ends^69^. Analysis was performed by GenXPro GmbH (Frankfurt, Germany). For that purpose, the MACE-kit v.2 was run according to GenXPro GmbH. To that end, RNA was fragmented and enriched polyadenylated mRNA underwent poly-A specific reverse transcription and template-switch-based second strand syntheses followed by competitive PCR. Duplicate reads verified by implemented unique molecular identifiers (TrueQuant IDs) were eliminated from the raw dataset. Using cutadapt (https://github.com/marcelm/cutadapt/), low-quality sequence bases were eliminated. Poly(A)-tails were clipped by an in-house Phython-Script. After mapping the reads on the mouse reference genome (mm10), transcripts were quantified by HTSeq. Differentially expressed genes were identified by DESeq2^70^.

### In situ hybridization by RNA scope

In order to analyze C5aR1 mRNA expression in liver tissue the RNAscope in situ hybridization technique was performed (Advanced Cell Diagnostics (ACD), Newark, CA, USA) by using a specific probe, namely Mm-C5aR1 (ACD, 439951). 4 µm paraffin sections were deparaffinized and treated with H_2_O_2_ for 10 min at room temperature. Antigen retrieval was performed for 30 min followed by protease treatment for 30 min. The probe was hybridized for 2 h followed by all incubations according to the RNAscope® 2.5.HD Detection Reagent – BROWN (ACD 322310).

### Statistical analysis

Data were first evaluated with the Kolmogorow–Smirnow-test for parametric distribution. For comparison of two groups, raw data were analyzed by unpaired two-tailed Student’s *t*-test or by a Mann–Whitney-U-test. For comparison of three or more groups, raw data were analyzed by one-way ANOVA with post hoc Bonferroni correction or by the Kruskal–Wallis test followed by Dunn’s post hoc test—as indicated in the figure legends. Throughout the manuscript, ‘n-counts’ refer to individual mice evaluated in the given experimental setup. Data are shown as means ± s.e.m. or as box-plots (with the top and bottom margins describing the 75th and 25th percentile, with whiskers depicting maximum and minimum values and with a horizontal line indicating the median; potential outliers, determined by ±1.5x IQR, are included in statistical analyses (Supplementary Fig. 1a-right, b–left panel, c-left panel; Supplementary Fig. 2d)) and are presented as fold-induction (*versus* control), as (% of total liver section), or as raw data (target gene expression *versus* GAPDH, U/L, cleaved Caspase-3 (+)-nuclei counts/necrotic area representing 1% of liver section, PCNA (+) nuclei per field of vision). In case an experimental group consists of ‘*n* ≤ 10’, all data points of the subfigure are shown. Differences were considered statistically significant if the *p*-value was below 0.05 (GraphPad Prism 8, CA, USA).

### Reporting summary

Further information on research design is available in the Nature Research Reporting Summary linked to this article.

## Supplementary information

Supplementary Figures

Reporting Summary

## Data Availability

The data sets generated and analyzed during this study are available from the corresponding author on reasonable request. Sequencing (MACE) data (Figs. 1d and 2) were deposited in NCBI’s Gene Expression Omnibus (GEO) under the accession number GSE169071.
