# Supplementary material for: 3′mRNA sequencing reveals pro-regenerative properties of c5ar1 during resolution of murine acetaminophen-induced liver injury
Source: NPJ Regen Med. 2022 Jan 27;7:10. doi: 10.1038/s41536-022-00206-x (PMC8795215; doi:10.1038/s41536-022-00206-x)
Supplement: Supplementary file 1 — Supplementary Figures [file 41536_2022_206_MOESM1_ESM.pdf]

Supplementary Figure 1

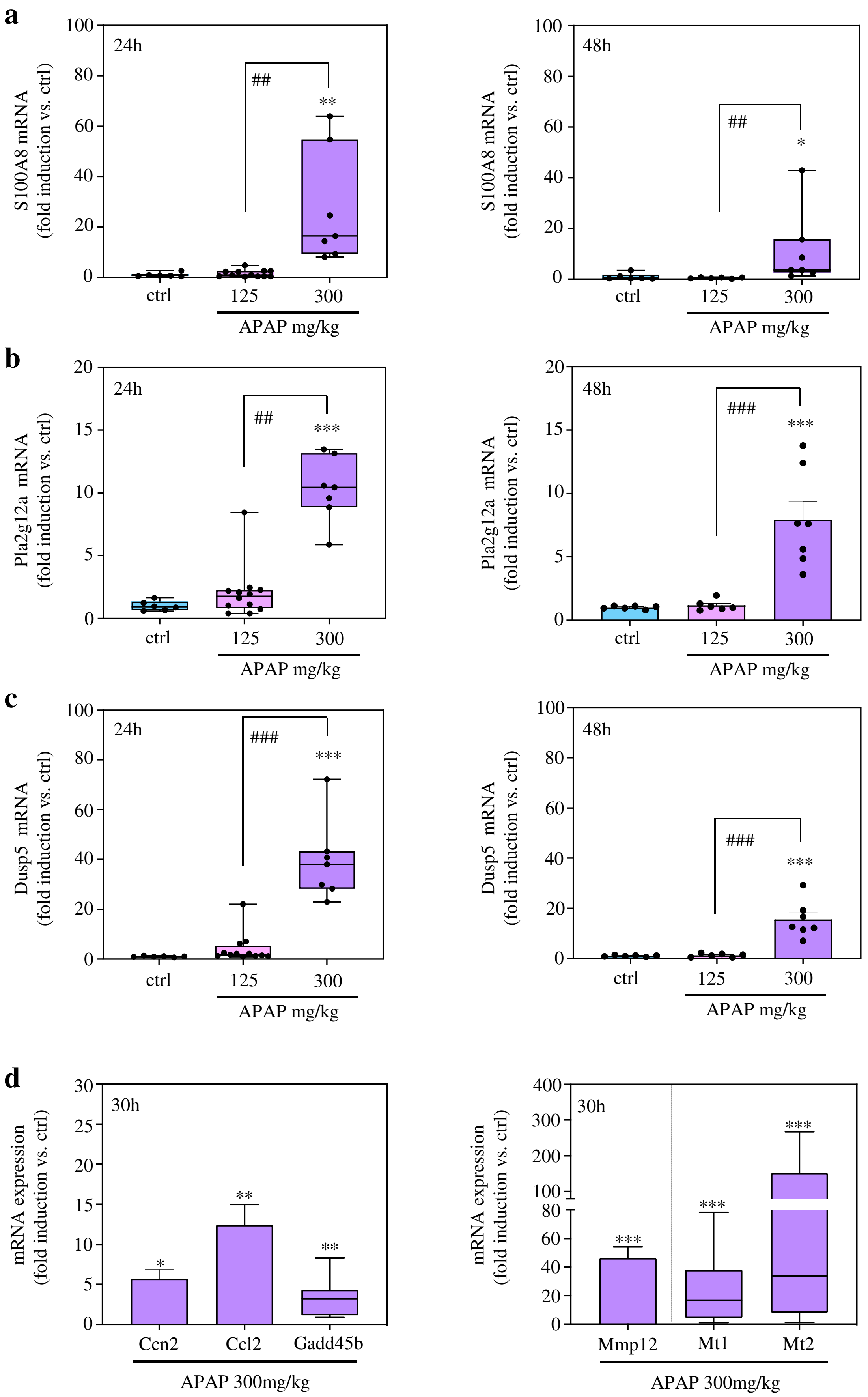

Supplementary Figure 1 Verification of MACE data by realtime PCR performed on individual hepatic RNA samples. a, b, c C57BL/6J mice received 0.9% NaCl (n = 6), APAP at 125 mg/kg (n = 12 for 24h, n = 6 for 48h), or APAP at 300 mg/kg (n = 7). After 24h/48h, hepatic RNA was isolated. For controls and APAP (300 mg/kg), individually analyzed RNA populations were the same that were used for pooling in MACE. Hepatic S100A8 (**a**), Pla2g12a (**b**), and Dusp5 (**c**) mRNA was determined (realtime PCR) 24h (left panel) or 48h (right panel) after APAP administration. Target mRNA normalized to GAPDH is shown as fold-induction compared the control of the same time point (*p < 0.05, **p < 0.01, ***p < 0.001 vs. control at the same time point; $^{\#\#}$p < 0.01, $^{\#\#\#}$p < 0.001). **d** C57BL/6J mice received 0.9% NaCl (n = 7) or APAP at 300 mg/kg (n = 11). After 30h, hepatic RNA was isolated. mRNA for Ccn2, Ccl2, Gadd45b (left panel), Mmp12, Mt1, and Mt2 (right panel) was determined by realtime PCR. Target mRNA normalized to GAPDH is shown as fold-induction compared to control (*p < 0.05, **p < 0.01, ***p < 0.001 vs. control). Statistical analysis on raw data: (**a** and **b-, c-**left panel) Kruskal-Wallis test with Dunn's post hoc test, (**d** Gadd45b, Mt1/2) Mann-Whitney-U-test, data are shown as box-plots; (**b-, c**-right panel) ANOVA with Bonferroni post hoc test; (**d** Ccn2, Ccl2, Mmp12) Student's *t*-test; data are shown as means ± SEM.

Supplementary Figure 2

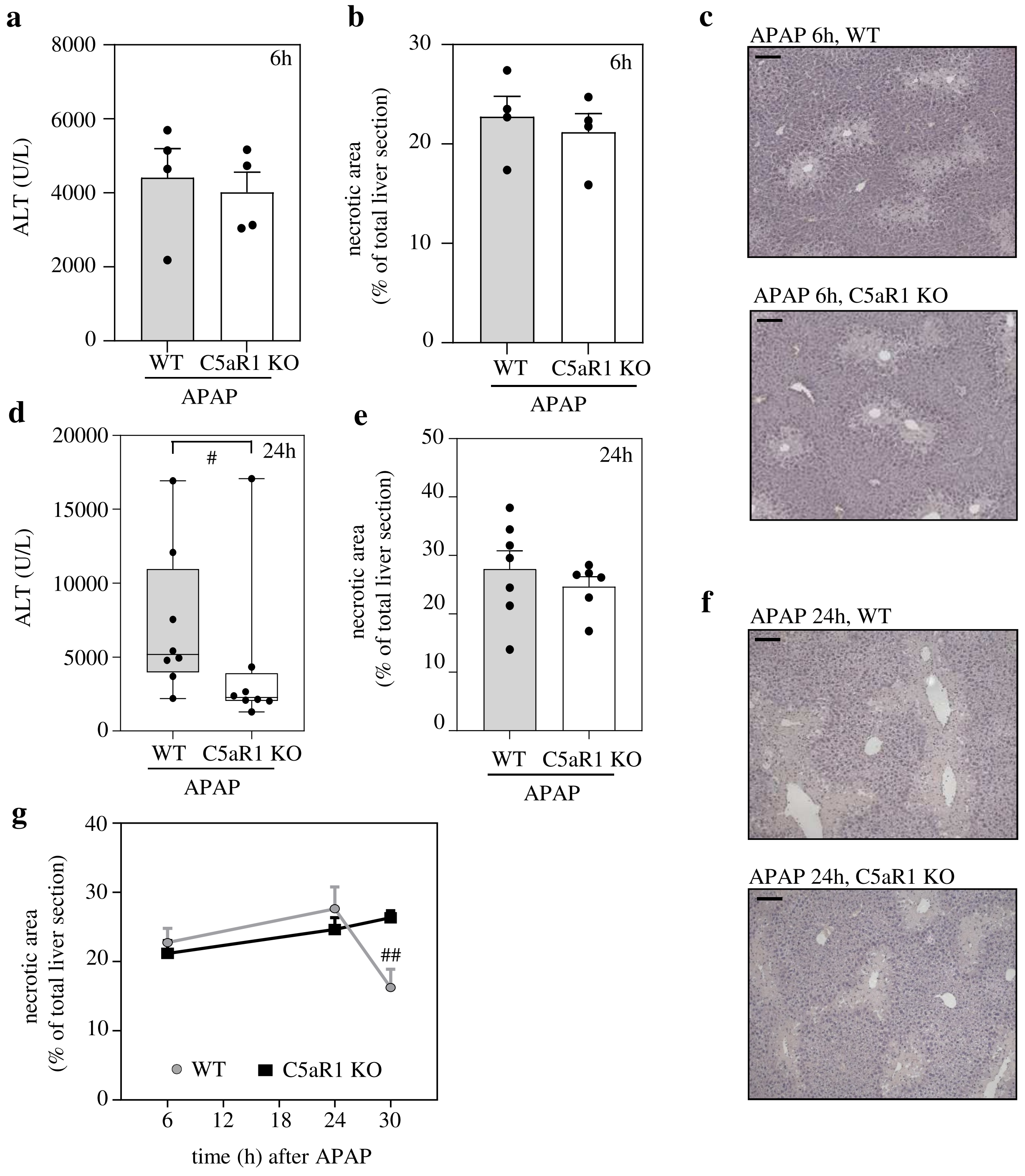

Supplementary Figure 2 C5aR1-deficient mice do not display enhanced liver injury at 6h and 24h after APAP administration. C57BL/6J C5aR1-deficient mice and their wild-type counterparts received APAP at 300 mg/kg. After 6h (**a - c, g**), 24h (**d - f, g**), or 30h (**g**), liver tissue and sera were analyzed. **a, d** Liver damage as detected by serum ALT (**a**, wt and c5ar1$^{-/-}$: n = 4; **d**, wt and c5ar1$^{-/-}$: n = 8; #p < 0.05). **b, e, g** Analysis by BZ-II analyzer software of necrotic areas in H&E-stained liver sections from APAP treated mice (**b**, wt and c5ar1$^{-/-}$: n = 4; **e**, wt: n = 7 and c5ar1$^{-/-}$: n = 6; **g**, 6h/24h see **b/e** and 30h: wt, n = 11 and c5ar1$^{-/-}$, n = 12; ##p < 0.01 (for WT 30h APAP vs. WT 24h APAP and for WT 30h APAP vs. c5ar1$^{-/-}$ 30h APAP)). **c, f** Representative liver sections analyzed by H&E staining at 6h or 24h (**c**, wt and c5ar1$^{-/-}$: n = 4; **f**, wt = 7 and c5ar1$^{-/-}$, n = 6). Statistical analysis on raw data: **a, b, e** Student`s *t*-test, data are shown as means ± SEM; **d** Mann Whitney-U-test, data are shown as box-plots; **g** Anova with Bonferroni post hoc test, data are shown as means ± SEM. Wild-type mice, WT; knockout mice, KO. Scale bars: 50 µm.
