# Supplementary material for: 3′mRNA sequencing reveals pro-regenerative properties of c5ar1 during resolution of murine acetaminophen-induced liver injury
Source: NPJ Regen Med. 2022 Jan 27;7:10. doi: 10.1038/s41536-022-00206-x (PMC8795215; doi:10.1038/s41536-022-00206-x)
Supplement: Supplementary file 2 — Reporting Summary [file 41536_2022_206_MOESM2_ESM.pdf]

nature research

Corresponding author(s): Heiko Mühl

Last updated by author(s): Dec 3, 2021

Reporting Summary

Nature Research wishes to improve the reproducibility of the work that we publish. This form provides structure for consistency and transparency in reporting. For further information on Nature Research policies, see our Editorial Policies and the Editorial Policy Checklist.

Statistics

For all statistical analyses, confirm that the following items are present in the figure legend, table legend, main text, or Methods section.

n/a	Confirmed	
☐	☒	The exact sample size (*n*) for each experimental group/condition, given as a discrete number and unit of measurement
☐	☒	A statement on whether measurements were taken from distinct samples or whether the same sample was measured repeatedly
☐	☒	The statistical test(s) used AND whether they are one- or two-sided<br>*Only common tests should be described solely by name; describe more complex techniques in the Methods section.*
☒	☐	A description of all covariates tested
☐	☒	A description of any assumptions or corrections, such as tests of normality and adjustment for multiple comparisons
☐	☒	A full description of the statistical parameters including central tendency (e.g. means) or other basic estimates (e.g. regression coefficient) AND variation (e.g. standard deviation) or associated estimates of uncertainty (e.g. confidence intervals)
☐	☒	For null hypothesis testing, the test statistic (e.g. *F*, *t*, *r*) with confidence intervals, effect sizes, degrees of freedom and *P* value noted<br>*Give P values as exact values whenever suitable.*
☒	☐	For Bayesian analysis, information on the choice of priors and Markov chain Monte Carlo settings
☒	☐	For hierarchical and complex designs, identification of the appropriate level for tests and full reporting of outcomes
☒	☐	Estimates of effect sizes (e.g. Cohen's *d*, Pearson's *r*), indicating how they were calculated

Our web collection on statistics for biologists contains articles on many of the points above.

Software and code

Policy information about availability of computer code

Data collection	3'mRNA sequencing (GenXPro): HTSeq, DESeq.<br>Real-time PCR: SDS v1.4 (7500 Fast Systeme , Thermo Fisher Scientific) and Design & Analysis Software v1.4.3 (QS3, Thermo Fisher Scientific)
Data analysis	Quantification of histological liver injury: Keyence BZ-II Analyzer software H2AE<br>Statistics and design of figures: GraphPad Prism v8.4.2

For manuscripts utilizing custom algorithms or software that are central to the research but not yet described in published literature, software must be made available to editors and reviewers. We strongly encourage code deposition in a community repository (e.g. GitHub). See the Nature Research guidelines for submitting code & software for further information.

Data

Policy information about availability of data

All manuscripts must include a data availability statement. This statement should provide the following information, where applicable:

- Accession codes, unique identifiers, or web links for publicly available datasets
- A list of figures that have associated raw data
- A description of any restrictions on data availability

The data sets generated and analyzed during this study are available from the corresponding author on reasonable request. Sequencing (MACE) data (Fig. 1d, Fig. 2) were deposited in NCBI's Gene Expression Omnibus (GEO) under the accession number GSE169071.

Field-specific reporting

Please select the one below that is the best fit for your research. If you are not sure, read the appropriate sections before making your selection.

☒ Life sciences ☐ Behavioural & social sciences ☐ Ecological, evolutionary & environmental sciences

For a reference copy of the document with all sections, see nature.com/documents/nr-reporting-summary-flat.pdf

Life sciences study design

All studies must disclose on these points even when the disclosure is negative.

Sample size	Sample sizes agree with the authors' previous experience concerning experimental acetaminophen-induced liver injury. Initially, animal numbers were estimated based on the ability to detect treatment- and/or genotype-dependent effects of approx. 30-40% (with $p < 0.05$). Sample sizes may vary depending on animal availability.
Data exclusions	No data were excluded from the study.
Replication	3'mRNA sequencing was performed once with pooled samples (as outlined in the Methods section).<br>For in vivo experiments, the numbers of replicates (= mice) are indicated in the figure legends.
Randomization	Mice (wt or c5aR1-ko) were randomly assigned to control- or treatment-groups.
Blinding	IHC studies were analyzed in blinded manner.

Reporting for specific materials, systems and methods

We require information from authors about some types of materials, experimental systems and methods used in many studies. Here, indicate whether each material, system or method listed is relevant to your study. If you are not sure if a list item applies to your research, read the appropriate section before selecting a response.

Materials & experimental systems

n/a	Involved in the study	
☐	☒	Antibodies
☒	☐	Eukaryotic cell lines
☒	☐	Palaeontology and archaeology
☐	☒	Animals and other organisms
☒	☐	Human research participants
☒	☐	Clinical data
☒	☐	Dual use research of concern

Methods

n/a	Involved in the study	
☒	☐	ChIP-seq
☒	☐	Flow cytometry
☒	☐	MRI-based neuroimaging

Antibodies

Antibodies used	1) Polyclonal (rabbit) antibody detecting (human, rat, murine) active (cleaved) caspase-3<br>(Innovative Diagnostik Systeme, Hamburg, Germany; #CI752C002).<br><br>2) Monoclonal (rabbit) antibody detecting (human, rat, murine, monkey) PCNA (D3H8P)<br>(Cell Signalling, Frankfurt, Germany; #13110)
Validation	1) #CI752C002 was tested by the supplier and successfully used in published studies analyzing murine tissues:<br>- Goren I et al. Systemic anti-TNFalpha treatment restores diabetes-impaired skin repair in ob/ob mice by inactivation of macrophages. J Invest Dermatol. (2007) 127:2259-67.<br>- Strüh CM et al. Triterpenoids amplify anti-tumoral effects of mistletoe extracts on murine B16.f10 melanoma in vivo. PLoS One (2013) 8:e62168.<br><br>2) #13110: Validation and references see on<br>https://www.cellsignal.de/datasheet.jsp?productId=13110&images=1

Animals and other organisms

Policy information about studies involving animals; ARRIVE guidelines recommended for reporting animal research

Laboratory animals	All mice used herein were male from the C57BL/6J strain (9–12 weeks old). Mice were maintained under a 12h light-dark cycle in

Laboratory animals	type II-long-IVC with access to food and water ad libitum - with the exception of a 10h overnight fasting period (with free access to water) before APAP (or vehicle) administration. For details, see the Methods section.
Wild animals	The study did not involve wild animals.
Field-collected samples	The study did not involve samples collected from the field.
Ethics oversight	All animal experiments (male C57BL/6J mice, 9–12 weeks old) were carried out in accordance with the recommendations of the Animal Protection Agency of the Federal State of Hessen (Regierungspräsidium Darmstadt, Germany) and were approved by the Regierungspräsidium Darmstadt (references V54-19c20/15-FU1190 and -FU1230).

Note that full information on the approval of the study protocol must also be provided in the manuscript.
